# Artherial thrombosis with antiphospholipid syndrome in 17 year old female patient: case of familial antiphospholipid syndrome

**DOI:** 10.1186/1546-0096-9-S1-P217

**Published:** 2011-09-14

**Authors:** M Vidović, S Dobrota, D Perkov, Lj Banfić, K Putarek, K Starčević, M Jelušić-Dražić

**Affiliations:** 1 Department of Paediatrics, Division of Paediatric Rheumatology and Immunology, University Hospital Center Zagreb, Croatia; 2 Department of Diagnostic and Interventional Radiology, University Hospital Center Zagreb, Croatia; 3 Department of Cardiovascular Disease, University Hospital Center Zagreb, University of Zagreb School of Medicine, Croatia

## Background

The Antiphospholipid syndrome is a multiorgan, autoimmune disorder characterized by repeated vascular thrombosis, pathologic pregnancies, thrombocytopenia and positive antiphospholipid antibodies (aPL), anticardiolipin antibodies (aCL) and/or anti-ß2 glycoprotein I antibodies (anti-ß2GP I). There is primary and secondary antiphospholipid syndrome, «catastrophic» and so called «familial”APS connected to HLA gene loci DR 4, DR 7, DQB1 i DRB 1 and valin247/leucin polymorphism on antigen for ß2GP I.

This case report will present 17-year old, previously healthy girl admitted to our Department of Paediatrics, Division of Paediatric Rheumatology, with occlusion of right radial and left popliteal artery. She used combined progesterone-estrogen contraceptive in August 2009 for few months because of irregular menstrual cycle. The illness started a month before admittance with rash and itching of right hand and left foot accompanied with pain in left lower leg. She was initially hospitalized in local hospital where she was diagnosed with right radial and left popliteal arterial occlusion and treated unsuccessfully with low molecular heparin. After she was transferred to our Divison mid December 2009 for further consultations and treatment she developed clinical symptoms of complete left popliteal occlusion. She was transferred to Department of Cardiovascular Disease where she successfully underwent fibrinolysis with alteplase. Laboratory findings showed increased ß2GP I and cardolipin antibodies measures on admittance and after 12 weeks period which led us to antiphspholipid syndrome diagnosis. In the mean time patients mother developed deep vein thrombosis of lower extremities and is in process of further immunologic testing, so it could be a case of familial antiphospholipid syndrome.

## Conclusion

We described a case of 17 year old girl with critical occlusion of left popliteal and right radial artery treated unsuccessfully with low-molecular heparin and underwent fibrinolysis with alteplase. Twelve weeks after hospitalization she still had positive anticardiolipin antibodies and is on anticoagulation therapy, since her mother had deep vein thrombosis with positive antiphospholipid antibodies there is a possibility of familial occurrence of antiphospholidi syndrome.

